# The Mediating Effect of Perceived Occupational Stress and Job Satisfaction on the Impact of Type D Personality on Turnover Intention Among Chinese General Practitioners

**DOI:** 10.3390/healthcare14121713

**Published:** 2026-06-15

**Authors:** Minghe Xu, Hairong Zhou, Erya Wen, Jian Yang, Chuanan Wu, Weiqing Chen

**Affiliations:** 1 Department of Epidemiology, School of Public Health (Shenzhen), Sun Yat-sen University, Shenzhen 515100, China; xumh37@mail2.sysu.edu.cn (M.X.); wenery3@mail2.sysu.edu.cn (E.W.); 2 College of General Practice, Southern University of Science and Technology, Shenzhen 515100, China; 13418850411@139.com (H.Z.); 15170345215@139.com (J.Y.)

**Keywords:** perceived occupational stress, job satisfaction, Type D personality, turnover intention, general practitioners

## Abstract

**Highlights:**

**What are the main findings?**

- Type D personality is significantly positively associated with turnover intention among Chinese general practitioners (GPs).
- Under the hypothesized sequential model, perceived occupational stress and job satisfaction accounted for 30.68% of the total covariance between Type D personality and turnover intention.

**What are the implications of the main findings?**

- Managers of Chinese community healthcare centers may benefit from paying particular attention to general practitioners with Type D personality, as they appear more vulnerable to occupational stress and lower job satisfaction.
- Efforts to reduce perceived occupational stress and improve job satisfaction may be considered as possible pathways to mitigate turnover intention among Type D personality GPs and support primary healthcare workforce stability.

**Abstract:**

Background/Objectives: General practitioners (GPs) face critical workforce shortages and high turnover globally. While external factors are known to influence turnover intention (TI), the role of individual psychological traits is less well understood. This study examines the association between Type D personality (TDP) and TI among GPs and the co-occurring statistical associations of perceived occupational stress (POS) and job satisfaction (JS). Methods: A cross-sectional survey was conducted from September to October 2024 among 383 GPs in Longhua District, Shenzhen, China. Participants completed a structured questionnaire assessing socio-demographic characteristics, TDP, POS, JS, and TI. After controlling potential confounders, correlation and regression analyses were performed to assess associations between TDP, POS, JS, and TI. Structural equation modeling (SEM) was conducted to examine the specific indirect associative components within the covariance between TDP and TI. Results: After adjusting for confounding factors, TDP was significantly positively associated with TI (B = 0.71) and POS (B = 0.30), and significantly negatively associated with JS (B = −0.24). In the hypothesized structural model, the proportions of total standardized covariance attributable to the indirect associative paths involving POS alone, JS alone, and the serial combination of POS and JS were 17.28%, 9.90%, and 3.50%, respectively, summing to 30.68% of the model-implied association. Conclusions: GPs with TDP reported a higher level of turnover intention, and this association was statistically accompanied by elevated occupational stress and diminished job satisfaction. Healthcare managers may consider implementing targeted interventions aimed at reducing stress and enhancing satisfaction, particularly among GPs with TDP, although the effectiveness of such strategies requires confirmation in future longitudinal or intervention studies.

## 1. Introduction

General practitioners (GPs), as the core workforce of the primary healthcare system and “gatekeepers” of community health, play critical roles including the diagnosis and treatment of common illnesses, coordination of referrals, preventive care, chronic disease management, and integrated healthcare services [1,2,3,4]. Despite their indispensable role in the healthcare system, GPs persistently face numerous unresolved challenges around the world. Taking China as an example, the number of GPs in mainland China increased from 109,794 in 2012 to 434,868 in 2021, with 3.08 GPs per 10,000 people in 2021. The Gini coefficient of GPs allocation by population in China decreased from 0.312 to 0.147 from 2012 to 2021, while the Gini coefficient of geographic dimension remained between 0.700 and 0.750 [5], which indicated a significant gap compared to developed countries. Regarding working conditions and professional environment, Chinese GPs also face multiple challenges. GPs’ salaries are generally low, significantly less than those of specialist doctors at the same level [6,7]. In addition, there is low social recognition of the profession, and the public often lacks trust in primary healthcare institutions and tends to seek health care directly in large hospitals [8,9]. These factors—low salary, lack of social recognition, and insufficient institutional support—contribute to widespread job dissatisfaction and high turnover intentions among GPs in China. For example, a survey reported that up to 70.0% of GPs expressed a strong intention to leave their jobs [10].

Turnover intention (TI), defined as an individual’s subjective expectation of leaving their current position or organization in the future, is widely recognized as the most effective predictor of actual turnover behavior [11,12]. Turnover intention is affected by both occupational and psychological factors, including personality traits, work environment, occupational stress, job satisfaction, etc. [13,14]. Perceived occupational stress (POS) refers to the state of tension or threat experienced by practitioners in their professional activities, and excessive stress can negatively impact their job performance and quality of life [15]. A study showed that the lower perceived occupational stress could reduce turnover intention among intensive care unit nurses [16]. Job satisfaction (JS) refers to the positive emotional experience derived from an individual’s evaluation of job characteristics [17], and extensive research confirmed it has a significant negative correlation with turnover intention [18]. For example, several studies showed that improving job satisfaction might reduce physicians’ turnover intention [19,20]. Furthermore, the close relationship between occupational stress and job satisfaction has become a consensus in academia [21,22,23]. Personality generally determines how a person reacts to things, how they communicate with other people, and their emotions, thoughts, and behaviors [24]. Therefore, even if individuals are exposed to the same occupational stressor, their responses may differ, which suggests that personality traits may play a moderating role in stress appraisal and coping strategy selection, thereby influencing career adaptation outcomes [23]. Existing research has initially revealed the association between occupational stress and personality dimensions. For example, Subburaj et al. revealed a direct and significant correlation between job stress and five personality factors [25], and Sahar J et al. found a positive correlation between neuroticism and occupational stress that indicated that individuals being prone to emotional instability were more likely to experience stress [26].

**Figure 1:**
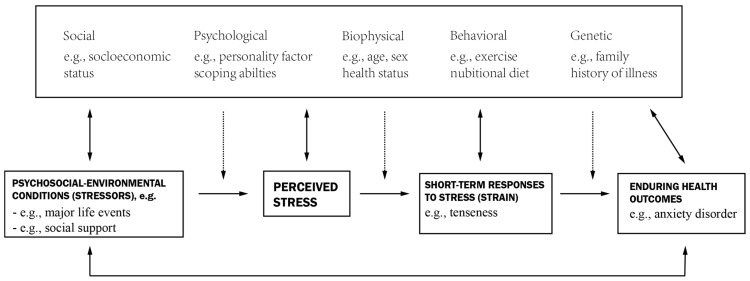
Occupational Stress Process Model (Simplified). Conceptual framework of the stress process (French & Kahn, 1962; House, 1981; Israel, Schurman, & House, 1989; Israel & Schurman, 1990; Katz & Kahn, 1978). Solid lines between boxes indicate presumed relationships among variables. Dotted lines indicate the hypothesized buffering effects of the modifying variables on the relationship between stressors and perceived stressors, between perceived stress and short-term responses, and between short-term responses and enduring health outcomes. From Figure 1 of “Action Research on Occupational Stress: Involving Workers as Researchers,” by B. A. Israel, S. J. Schurman, and J. S. House, 1989, p. 137 [28]. Copyright 1989 by Baywood Publishing Co. Adapted with permission.

Traditional personality typologies, such as the Type A/B/C/D classification, have been used to understand disease-prone and health-related behaviors. Type A (competitive, time-urgent) and Type B (relaxed) were initially studied in cardiovascular contexts; Type C (cancer-prone, emotionally suppressed) has been examined in oncology research. Type D (also termed ‘distressed personality’), in contrast, is specifically characterized by the co-occurrence of negative affectivity and social inhibition, and has been increasingly recognized as a relevant personality construct in occupational health because it amplifies stress perception and is associated with adverse work outcomes. We chose the Type D concept because it directly maps onto the stress appraisal and coping pathways postulated in the Occupational Stress Process Model, and because prior studies have demonstrated its predictive utility for burnout and turnover cognitions in healthcare professionals. As an important individual personality trait, Type D personality (TDP) refers to individuals who exhibit high levels of both negative affectivity (NA) and social inhibition (SI) [27]. Guided by the theoretical framework of the Occupational Stress Process Model [28], individual personality characteristics have been recognized as relevant factors that may relate to the appraisal of occupational stressors, and may further correlate with subsequent psychological and behavioral responses, and ultimately show statistical associations with occupational behavioral outcomes such as turnover intention (Figure 1). According to this model, occupational stress is a dynamic and multi-stage process in which objective stressors, perceived stress, short-term responses, and long-term outcomes are interconnected through a series of feedback loops. Crucially, the model identifies modifying factors—including individual psychological traits—that can shape how stressors are perceived and experienced. Within this framework, Type D personality can be conceptualized as a stable modifying factor at the individual level. The model posits a sequential logic: modifying factors are associated with perceived stress, which in turn is associated with short-term psychological and behavioral responses, which may ultimately be associated with long-term occupational outcomes such as turnover intention. This theoretical sequence—modifying factors → perceived stress → short-term responses → long-term outcomes—closely aligns with the variables examined in the present study: Type D personality (modifying factor), perceived occupational stress (perceived stress), job satisfaction (short-term response), and turnover intention (long-term outcome).

Existing empirical studies have discovered the close link between TDP, occupational stress responses and occupational outcomes: for instance, Renzo showed through correlation, multiple regression and relative weight analysis that burnout is mainly related to neuroticism [29], while Jahan Bakhsh et al. further pointed out that physicians with TDP faced seven times the risk of burnout compared to non-Type D individuals [30]. In fact, the influence of personality on occupational health through psychological and behavioral pathways has been established as a proposition supported by substantial evidence [31]. As a key pillar in establishing a tiered healthcare system and deepening healthcare reform, the stability of the GP workforce is directly linked to the rational operation and service efficiency of the entire healthcare system. Therefore, systematically investigating the causes of turnover and developing retention strategies are urgent practical issues.

Notably, this study adopted a predictor–intermediary–outcome associative framework for statistical covariance decomposition. TDP was treated as a predictor variable rather than a moderator/modifying factor in the classic Occupational Stress Process Model. The current framework is theoretically informed but not identical to the original model; thus, all results should be interpreted as cross-sectional statistical associations rather than causal stress processes.

**Figure 2:**
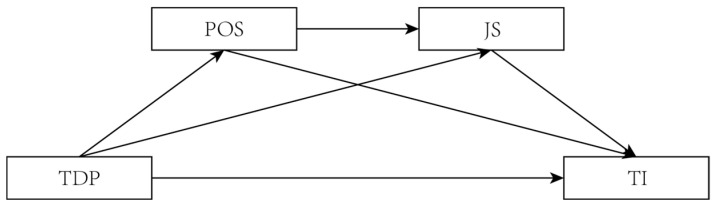
Hypothetical model.

Against this theoretical and empirical background, this study aimed to examine the statistical associations of TDP with turnover intention among GPs in Shenzhen, China, and to explore the potential statistical intervening roles of perceived occupational stress and job satisfaction from an individual psychological perspective. Based on the above theoretical and empirical evidence, we proposed the following hypotheses (Figure 2, hypothetical model):

**H1.**  *TDP may positively correlate with GPs’ TI.*

**H2.**  *TDP may positively correlate with GPs’ POS.*

**H3.**  *TDP may negatively correlate with GPs’ JS.*

**H4.**  *POS and JS show statistical indirect associative components in the association between TDP and TI.*

## 2. Materials and Methods

### 2.1. Participants

We conducted a cross-sectional survey at People’s Hospital of Longhua District in Shenzhen, China. This survey was designed to investigate the relationship between Type D personality and turnover intention. The inclusion criteria were as follows: (1) informed consent and voluntary participation in the survey and (2) more than 3 months of service. The exclusion criterion was (1) being an intern or administrator. All information provided by the participants was kept confidential and gave their informed consent before completing the questionnaire.

### 2.2. Data Collection

From October to November 2024, convenience sampling was used to recruit participants via the hospital’s internal WeChat platform to complete a structured questionnaire, which assessed their sociodemographic characteristics, Type D personality, perceived occupational stress, job satisfaction, and turnover intention. Before accessing the questionnaire, participants were provided with an electronic information sheet explaining the study’s purpose, voluntary nature of participation, confidentiality protections, and withdrawal rights. Participants could proceed to the survey only after providing electronic informed consent. A total of 383 questionnaires were returned, all of which were valid (validity rate: 100%). The response rate among invited participants (i.e., those who opened the link) was 100%.

### 2.3. Type D Personality Measurement

The Type D Scale-14 (DS-14) scale was selected as the measurement tool for Type D personality, which was designed by Denollet and has been proven to have good reliability and validity [32]. The DS-14 consists of 14 items divided into two dimensions: negative affectivity (NA) and social inhibition (SI), each containing seven items. The scale uses a 5-point Likert scale (0 = “not at all true” to 4 = “very true”), with items 1 and 3 being reverse-scored. Higher scores indicate more pronounced Type D personality traits. In this study, the DS-14 scale demonstrated good internal consistency (Cronbach’s α = 0.864).

In this study, Type D personality was operationalized in two complementary ways, in line with its theoretical definition as a configural trait (the co-occurrence of high NA and high SI). First, we used the traditional dichotomous classification for descriptive and supplementary analyses, wherein Type D personality is defined when both NA and SI dimension scores are ≥10. This classification is the most widely used standard in clinical and occupational health screening practice globally and facilitates the preliminary translation of our findings into practical occupational health monitoring protocols. Second, for our primary structural equation modeling (SEM) analyses, we modeled Type D personality as a latent construct, with NA and SI as its observed indicators. This dimensional approach was adopted for both theoretical and methodological reasons. First, research has demonstrated that Type D personality is better characterized as a dimensional continuum rather than a discrete categorical entity [33]. Second, dichotomizing continuous personality dimensions into binary categories results in a substantial loss of statistical power and information, with estimates suggesting that up to 20–33% of variance may be discarded through such artificial categorization [26]. Third, latent variable modeling allows for the disattenuation of associations by separating true score variance from measurement error, thereby yielding less biased estimates of the indirect associative components under investigation [34]. Therefore, the dimensional approach is more appropriate for the present descriptive inquiry into the statistical associations among personality traits and occupational outcomes [35].

### 2.4. Perceived Occupational Stress Measurement

The perceived occupational stress scale used in this study was adapted from the Perceived Stress Scale-14 (PSS-14) [36], which consisted of 15 items and contained three dimensions: work recognition and rewards, work intensity and burden, work environment and interpersonal relationships. This scale uses a Likert-style format, with responses ranging from “not bothered at all” to “very bothered,” and scores ranging from 1 to 4. Higher scores indicate higher levels of perceived occupational stress. Confirmatory factor analysis (CFA) was conducted to examine the construct validity of the three-dimensional model. All standardized factor loadings ranged from 0.613 to 0.927 (all *p* < 0.001), showing satisfactory item validity. The composite reliability (CR) values of the three dimensions were 0.908, 0.921, and 0.917, exceeding the recommended threshold of 0.70. The average variance extracted (AVE) values were 0.622, 0.657, and 0.737, all above 0.50, supporting good convergent validity. Correlations among the three latent variables ranged from 0.675 to 0.793 (all *p* < 0.001) and were below 0.85, confirming acceptable discriminant validity. Overall, the measurement model demonstrated sound construct validity, and the scale’s Cronbach’s α coefficient was 0.918, indicating good reliability.

### 2.5. Job Satisfaction Measurement

Job satisfaction was measured using a 3-item scale developed by Cammann et al. [37], which consisted of three items: (1) “Overall, I dislike my job” (reverse-scored), (2) “Overall, I am satisfied with my job,” and (3) “Overall, I enjoy working here.” Respondents rated each item on a five-point Likert scale, with 1 representing “strongly disagree” and 5 representing “strongly agree”. The higher the score, the stronger the level of job satisfaction. In this study, the Cronbach’s α for the scale was 0.752.

### 2.6. Turnover Intention Measurement

Turnover intention was measured by a 3-item scale developed by Brough P [38]. The scale consists of three items: (1) the frequency of considering leaving the job in the past 6 months, (2) the likelihood of leaving in the next 6 months, and (3) the frequency of actively seeking new job opportunities. A 5-point Likert scale was used for scoring, ranging from “Never” to “Always,” with scores assigned from 1 to 5. Cronbach’s coefficient for this scale was 0.864.

### 2.7. Potential Confounders

The potential confounders in this study included sociodemographic (gender, education et al.) and job characteristics (job title, salary, working hours et al.). Empirical evidence showed that these characteristics were associated with job satisfaction and turnover intention [39].

### 2.8. Statistical Analysis

Because all variables were self-reported at a single time point, we conducted Harman’s single-factor test to assess common method bias. An unrotated principal component factor analysis was conducted using all items. If the variance explained by the first common factor is less than 40%, it suggests that common method bias is not a serious concern [40]. It should be noted that Harman’s single-factor test, as a post hoc passive method, only detects severe common method variance. It cannot eliminate all forms of common method bias or correct for the systematic measurement error arising from single-source self-report data.

Means and standard deviations (SD) were presented to describe continuous variables, while percentages were used to describe the categorical variables. The scale was tested for reliability and validity to optimize the scale structure and ensure data stability.

First, *t*-tests or one-way analysis of variance (ANOVA) were used to examine differences in turnover intention by demographic characteristics.

Second, we used regression analyses to examine how Type D personality, perceived occupational stress, and job satisfaction were associated with turnover intention, after controlling for potential confounders.

SPSS 26.0 statistical software was used for general description analysis, one-way analysis of variance, *t*-tests, and factor analysis. Structural equation modeling (SEM) was conducted to examine the specific indirect associative components within the covariance between Type D personality and turnover intention. The following fit indices were used to assess the overall model fit: the root mean square error of approximation (RMSEA), the goodness-of-fit index (GFI), the Tucker–Lewis index (TLI), the incremental fit index (IFI), and the comparative fit index (CFI). GFI, TLI, IFI, and CFI values above 0.90 and RMSEA value below 0.08 indicated acceptable fit [41].

## 3. Results

### 3.1. Common Method Bias Test

Harman’s single-factor test was employed to examine common method bias. The total variance explained by the first unrotated factor was 35.22%, which was below the critical threshold of 40%. Meanwhile, the KMO value was 0.937, and Bartlett’s test of sphericity was statistically significant (*p* < 0.001), indicating that the data were suitable for factor analysis. However, because all data were self-reported at a single time point, the potential influence of common method variance cannot be entirely eliminated.

### 3.2. Comparisons of Turnover Intention Among Different Socio-Demographics Characteristics

Table 1 describes the differences in turnover intention based on different sociodemographic characteristics. Significantly lower turnover intention was observed among GPs with higher professional titles and higher monthly income. However, no significant differences in turnover intention were found with respect to gender, age, marital status, or education level.

### 3.3. Association Between Type D Personality, Perceived Occupational Stress, Job Satisfaction and Turnover Intention

An association matrix of Type D personality, perceived occupational stress, job satisfaction and turnover intention is presented in Table 2. After controlling for the aforementioned confounders, Type D personality was significantly and positively associated with perceived occupational stress (B = 0.30), job satisfaction (B = −0.24) and turnover intention (B = 0.71), and perceived occupational stress was significantly and negatively associated with job satisfaction (B = −0.37) and turnover intention (B = 0.51), while job satisfaction was significantly and negatively associated with turnover intention (B = −0.51).

### 3.4. Indirect Associations Involving Perceived Occupational Stress and Job Satisfaction

Structural equation modeling (SEM) was performed to examine the statistical intervening associative components of POS and JS within the total covariance between TDP and TI. To ensure model parsimony, TDP, POS, JS, and TI were modeled as latent variables with their corresponding items as indicators. NA and SI were operationalized using their mean scores as observed variables. Mean scores of items or dimensions were used for other constructs. Guided by the hypothesized conceptual sequence of the Occupational Stress Process Model, four statistical associative components were specified: (1) a direct statistical path from TDP to TI; (2) an indirect statistical path from TDP to POS to TI; (3) an indirect statistical path from TDP to JS to TI; and (4) a serial indirect statistical path from TDP to POS to JS to TI.

**Figure 3:**
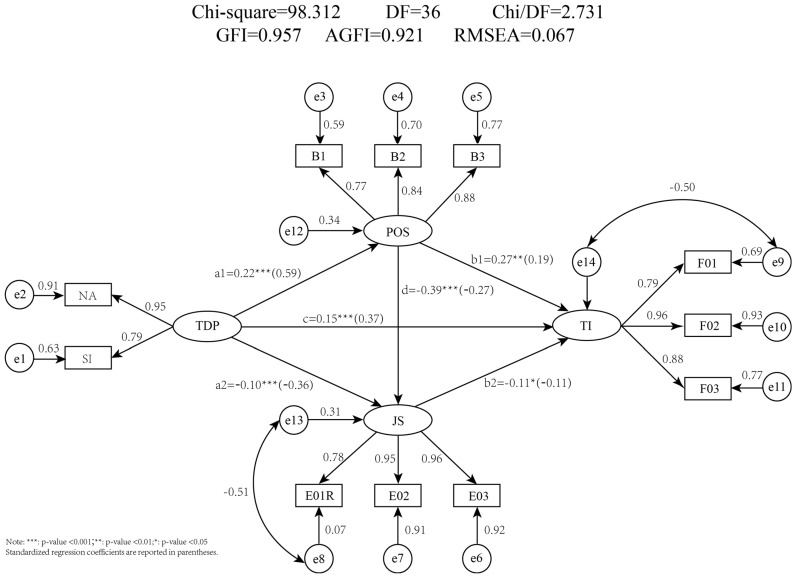
The standardized model of the relationship between TDP, POS, JS and TI among GPs. POS: perceived occupational stress; TDP: Type D personality; TI: turnover intention; JS: job satisfaction. B1–B3 are observed indicators of TDP; E01R, E02, E03 are observed indicators of POS (with E01R being a reverse-coded item); F01–F03 are observed indicators of TI. Ellipses represent latent variables, rectangles represent observed variables, single-headed arrows indicate causal paths, and double-headed arrows indicate correlations. e1–e14 denote measurement errors.

The SEM model fit indices were all within the quality standard range (GFI = 0.957, NFI = 0.968, CFI = 0.980, IFI = 0.980, TLI = 0.970, RMSEA = 0.067, RMR = 0.068, AGFI = 0.921), indicating an acceptable model fit. Under the hypothesized sequential model, the decomposition of covariance revealed one direct association and three specific indirect associations between TDP and TI (See Figure 3). First, TDP was positively associated with TI (c = 0.15). Second, TDP was positively associated with perceived occupational stress (a1 = 0.22), and POS was positively associated with turnover intention (b1 = 0.27). Third, TDP was negatively associated with job satisfaction (a2 = −0.10), and job satisfaction was negatively associated with turnover intention (b2 = −0.11). Fourth, TDP was positively associated with perceived occupational stress (a1 = 0.22) and was negatively associated with job satisfaction (d = −0.39); job satisfaction was negatively associated with turnover intention (b2 = −0.11).

Bias-corrected bootstrap testing estimated the magnitude and precision of the specific indirect associations (Table 3). In the hypothesized model, the direct association between TDP and TI was 0.357, comprising 69.32% of the total standardized covariance. The three specific indirect associations were as follows: 0.089 via POS alone (17.28%), 0.051 via JS alone (9.90%), and 0.018 via the serial combination of POS and JS (3.50%). The sum of these indirect associations was 0.158, representing 30.68% of the total model-implied covariance. It must be emphasized that these percentages reflect the decomposition of statistical association within the specified directional framework and should not be interpreted as evidence of causal mediation. The total standardized effect of TDP on TI was 0.515. Specifically, a one-SD increase in Type D score corresponds to a 0.15-SD increase in turnover intention, representing a small association with meaningful implications for workforce management.

### 3.5. Alternative Model Testing

To examine the robustness of the proposed sequential relationship (i.e., stress → satisfaction), an alternative model was estimated where job satisfaction preceded perceived occupational stress (i.e., TDP → JS → POS → TI). The results revealed that the alternative model yielded fit indices identical to those of the original model, indicating statistical equivalence between the two specifications. As a result, cross-sectional data alone are insufficient to disentangle which temporal ordering is more empirically tenable.

In such cases, model selection must be grounded in theoretical rationale. The sequential order of the original model (TDP → POS → JS) is consistent with the conceptual framework of the Occupational Stress Process Model, which posits a logical progression: from individual predisposing factor (i.e., Type D personality) to perceived stress, followed by short-term psychological responses (i.e., job satisfaction), and ultimately long-term occupational outcomes (i.e., turnover intention). Within this framework, perceived stress serves as the more proximal link associated with individual difference variables, whereas job satisfaction represents a downstream evaluative response shaped by, among other factors, the experience of stress. In contrast, the reverse ordering (TDP → JS → POS) lacks comparable theoretical support within this well-established theoretical model. Thus, despite their statistical equivalence, the original model is retained as the conceptually superior specification.

## 4. Discussion

To our knowledge, this is the first study to explore the statistical associations among Type D personality, perceived occupational stress, job satisfaction, and turnover intention within a sample of Chinese GPs. The results reveal several key patterns: Type D personality demonstrated significant positive associations with turnover intention and perceived occupational stress, and a significant negative association with job satisfaction. Furthermore, in the covariance decomposition analysis based on the hypothesized model, perceived occupational stress and job satisfaction accounted for a substantial proportion of the total covariance between Type D personality and turnover intention, with three distinct indirect associative components identified. All observed associations were directionally consistent with our a priori research hypotheses.

### 4.1. Interpretation of Sociodemographic Differences in Turnover Intention

Our bivariate analyses showed that GPs with intermediate professional titles and middle-level monthly income (¥15,000–20,000) reported significantly higher turnover intention. This pattern may reflect a career plateau effect for mid-career GPs, who face heavy workloads but limited promotion opportunities to senior titles due to stringent quotas. Regarding salary, this suggests a relative deprivation effect: lower-income GPs may be financially constrained from job change, whereas middle-income GPs earn enough to meet basic needs but insufficiently to compensate for high stress and low recognition, thereby increasing their turnover consideration.

### 4.2. Association Between Type D Personality and Perceived Occupational Stress

The finding that Type D personality is positively associated with POS aligns with a growing body of literature across various professional groups, including students and teachers [42,43,44,45,46], as well as physicians [30]. This finding has also been validated in broader occupational populations. For instance, a study among clinical nurses found that Type D personality was significantly associated with higher job stress and emotional exhaustion [47]. Similarly, in teacher samples, Type D personality was confirmed to be positively correlated with higher work stress and burnout symptoms [42]. Notably, a study among medical students demonstrated that individuals with Type D personality tend to adopt avoidance coping strategies when facing academic stress, thereby exacerbating their perceived stress [45]. Our study extends this observed association pattern to a sample of Chinese GPs. Within the framework of the Occupational Stress Process Model, Type D personality can be conceptualized as a stable individual predisposing factor (an individual-level psychological variable). According to this theoretical model, individual predisposing factors do not directly cause stress but are hypothesized to correlate with how objective work conditions are subjectively experienced. Individuals with high negative affectivity—a core component of TDP—are theorized to be predisposed to appraising work demands as more threatening or bothersome, which is consistent with the pattern of higher perceived stress observed among participants with elevated negative affectivity in this cross-sectional sample. This theoretical lens provides a coherent framework for interpreting the positive association observed between Type D traits and heightened POS among GPs in this study.

### 4.3. Association Between Type D Personality and Job Satisfaction

Consistent with prior research on physicians [30] and nurses [47], we observed a significant negative association between Type D personality and job satisfaction in this sample. The Occupational Stress Process Model posits that the stress process is dynamic, and that individual predisposing factors are hypothesized to correlate with not only the perception of stressors but also subsequent short-term psychological responses. In this theoretical framework, reduced job satisfaction can be viewed as a psychological response that correlates with the co-occurrence of high perceived stress and limited social resources, the latter being a characteristic of individuals with high social inhibition (the second component of TDP). The model further suggests that social inhibition may correlate with restricted access to supportive workplace relationships, which are widely recognized as crucial resources for maintaining positive work attitudes; this theoretical proposition aligns with the observed negative association between social inhibition and job satisfaction in our sample.

### 4.4. Association Between Perceived Occupational Stress and Job Satisfaction

The strong negative association between POS and job satisfaction observed in this study is consistent with one of the most robust and well-documented correlational patterns in occupational health psychology [48,49,50,51,52]. For example, a study among radiology technicians similarly demonstrated that higher perceived stress was associated with lower job satisfaction, and this association was independent of demographic variables such as age and years of service [51]. Among healthcare professionals, a Bayesian network analysis of Turkish healthcare workers also confirmed that occupational stress is a direct negative predictor of job satisfaction [52]. Furthermore, a cross-sectional study of rural health workers in 11 western provinces of China reported a similar negative correlation pattern [53]. Our study reaffirms this association among Chinese GPs. Within the sequential logic hypothesized in the Occupational Stress Process Model, this negative association aligns with the proposed theoretical progression from perceived stress to a short-term psychological response. These findings underscore the theoretical rationale for exploring interventions targeting the subjective experience of workplace stress, which may support both the mental health and job satisfaction of GPs, pending validation in future longitudinal and interventional research.

### 4.5. Association Between Perceived Occupational Stress, Job Satisfaction and Turnover Intention

Our findings are consistent with a vast body of literature documenting a positive association between POS and turnover intention [53,54,55,56,57,58,59], and a negative association between job satisfaction and turnover intention [4,53,60,61,62,63,64,65,66,67]. For instance, a study among Chinese emergency physicians found that occupational stress influenced turnover intention through the partial mediating role of depressive symptoms [55]. Among family doctors, work stress was also shown to directly or indirectly increase turnover intention [57]. Regarding job satisfaction, a national survey of village doctors in China indicated that job satisfaction was one of the strongest predictors of turnover intention [61]. Additionally, a study among hospital nurses in Shanghai similarly found that for every unit decrease in job satisfaction, turnover intention increased significantly [67]. In the context of the Occupational Stress Process Model, turnover intention is conceptualized as a long-term occupational outcome that is hypothesized to correlate with the accumulation of unresolved short-term strain responses, such as low job satisfaction, and the chronic experience of high perceived stress. The pattern observed in this sample—where higher stress correlates with greater intent to leave and higher satisfaction correlates with lower intent to leave—is entirely directionally consistent with this theoretical hypothesized sequence.

### 4.6. Indirect Associative Components via Perceived Occupational Stress and Job Satisfaction

Regarding the association between personality traits and turnover intention among professional workers, previous studies have identified significant associations between Big Five personality traits (e.g., neuroticism) and turnover intention in nursing and other healthcare professions [68,69]. However, few studies have examined the association between Type D personality and turnover intention among Chinese general practitioners (GPs). In this cross-sectional sample, our study found that Type D personality traits were significantly positively correlated with turnover intention among Chinese GPs. This finding is consistent with previous research results, which have suggested that individuals with TDP are more likely to adopt escape-avoidance coping strategies when facing occupational stressors, a pattern that correlates with increased cognitions around occupational withdrawal [42,45,70,71,72]. This observed association is also directionally consistent with the theoretical premise of the Occupational Stress Process Model, which posits that individual personality traits, as individual predisposing variables, may correlate directly with long-term occupational outcomes.

This theoretical model defines occupational stress as a complex and dynamic multi-stage process, with a core hypothesized logical chain: objective work environment stressors → individual perceived stress → short-term strain responses (physiological/psychological/behavioral) → long-term health and occupational outcomes. It also incorporates individual traits, psychosocial factors and other individual predisposing variables that are hypothesized to shape every link of the stress process, a framework that is highly compatible with the variable setting and observed associative findings of this study. Within this theoretical model framework, the variables in our study correspond to the following hypothesized roles: (1) individual predisposing variable: Type D personality (a personality trait variable at the individual level in the model); (2) perceived stress link: perceived occupational stress; (3) short-term psychological strain response: reduced job satisfaction; (4) long-term occupational outcome: turnover intention.

Based on this theoretical framework, we conducted structural equation modeling (SEM) to examine the statistical indirect associative components of POS and JS within the total covariance between TDP and TI. The results of the covariance decomposition showed that the total association between TDP and TI could be decomposed into one significant direct associative component (comprising 69.32% of the total model-implied covariance) and three significant indirect associative components (collectively accounting for 30.68% of the total association). All of these observed components are highly directionally consistent with the multi-stage stress process logic hypothesized in the Occupational Stress Process Model. The first indirect associative component, via POS alone, accounted for 17.28% of the total association. In the Occupational Stress Process Model, perceived stress is conceptualized as the core proximal construct hypothesized to connect objective stressors to subsequent strain responses and outcomes. The theoretical model posits that Type D personality (characterized by high negative affectivity and social inhibition), as an individual predisposing variable, may correlate with the association between objective occupational stressors and perceived occupational stress; this proposition aligns with the observation that GPs with higher Type D traits in this sample reported stronger subjective stress experience related to work stressors. This observed associative component directly corresponds to the core hypothesized path of “individual predisposing variable → amplified perceived stress → correlated long-term outcomes” in the theoretical model and was the indirect associative component with the largest standardized covariance in this study.

The second indirect associative component, via JS alone, comprised 9.90% of the total model-implied covariance. In the Occupational Stress Process Model, reduced job satisfaction is conceptualized as a short-term psychological strain response hypothesized to correlate with occupational stress, positioned as an intermediate construct between perceived stress and long-term occupational outcomes in the theoretical sequence. The theoretical model further posits that Type D personality, as an individual predisposing variable, may correlate not only with amplified perceived stress, but also with individuals’ cognitive evaluation and emotional experience of work; this aligns with the significant negative association observed between Type D personality and job satisfaction in our sample, as well as the well-documented negative association between job satisfaction and turnover intention. This observed associative component corresponds to the hypothesized path of “individual predisposing variable → correlated short-term psychological strain response →correlated long-term outcomes” in the theoretical model.

The third sequential indirect associative component via POS followed by JS comprised 3.50% of the total model-implied covariance. This observed associative pattern is fully directionally consistent with the core dynamic chain hypothesized in the Occupational Stress Process Model, which proposes that TDP, as an individual predisposing variable, correlates with amplified individual perceived occupational stress and is negatively correlated with job satisfaction, and ultimately correlates with higher turnover intention.

### 4.7. Differential Magnitude of Indirect Covariance via Perceived Occupational Stress Versus Job Satisfaction

Furthermore, we noted that in the hypothesized sequential model, the specific indirect associative component via perceived occupational stress comprised a larger proportion of the total model-implied covariance than the component via job satisfaction (17.28% versus 9.90%). While the statistical equivalence of the reversed model precludes definitive conclusions regarding temporal precedence, this differential pattern warrants cautious, exploratory interpretation within the theoretical framework guiding this study.

First, the structural positioning of variables within the theoretical model corresponds to the hierarchical difference in the magnitude of the observed statistical associations. According to the model, perceived stress is positioned as the core construct hypothesized to connect individual predisposing factors to subsequent responses in the conceptual sequence. Job satisfaction, by contrast, is positioned later in this sequence as a short-term psychological response. Type D personality, conceptualized as a stable individual predisposing factor in this framework, is characterized by high negative affectivity—a trait that is hypothesized to be associated with a heightened tendency to appraise work demands as threatening or bothersome [43]. Consistent with this proposition, individuals with higher Type D traits in this sample reported higher levels of perceived occupational stress. In this conceptual chain, POS is situated closer to the individual predisposing variable (TDP) in the model’s hypothesized sequence, whereas JS is positioned further downstream. Statistically, in cross-sectional covariance decomposition, an indirect associative component carried through a variable positioned earlier in the hypothesized conceptual sequence would be expected to account for a larger proportion of the total association than one positioned later.

Second, the characteristics of TDP align more closely with the stress-perception component of the theoretical model. TDP is defined by the co-occurrence of high negative affectivity and social inhibition. Previous studies have reported that individuals with TDP tend to report significantly higher levels of perceived stress than those without Type D traits when exposed to comparable objective work conditions, a pattern that has also been observed in samples of healthcare professionals. This suggests that TDP may share a more pronounced statistical association with perceived stress than with overall job evaluation. In other words, the covariance between TDP and turnover intention in this sample is more strongly aligned with the perceived stress appraisal component of the hypothesized model.

Third, the occupational context of Chinese general practitioners provides a fitting backdrop for this pattern of associations. Chinese GPs widely report exposure to multiple occupational stressors, including heavy workloads, limited remuneration, and insufficient social recognition. Surveys indicate that a substantial proportion of Chinese GPs report moderate-to-high levels of turnover intention. Within this context, occupational stress is consistently identified as a prominent correlate of turnover cognitions, whereas decreased job satisfaction often co-occurs with sustained high stress. Thus, in this sample, POS—as an indicator of perceived occupational stress—carries a larger portion of the indirect statistical association between TDP and turnover intention.

It must be reiterated, however, that these interpretations are entirely contingent upon the directional assumptions of the hypothesized model. The equivalent fit of the reversed model (TDP → JS → POS → TI) demonstrates that alternative temporal orderings are statistically indistinguishable with cross-sectional data. Consequently, the differential magnitude of these indirect covariance estimates should be viewed strictly as exploratory and hypothesis-generating, rather than as definitive evidence of differential effects. Longitudinal research is required to validate the temporal ordering of these constructs and to examine whether perceived stress indeed precedes and correlates more strongly with turnover intention than job satisfaction in the hypothesized sequence from TDP to turnover intention.

### 4.8. Recommendations and Implications

In summary, the present cross-sectional findings identify significant associations between Type D personality, perceived occupational stress, job satisfaction, and turnover intention among Chinese GPs, providing preliminary evidence to inform targeted, exploratory strategies for healthcare administrators and hospital managers. Several feasible, evidence-informed directions are summarized as follows:

Voluntary psychological assessment for mental health awareness: The DS-14 scale may be used as a voluntary, non-diagnostic, exploratory tool to help GPs gain general insight into TDP patterns and associated stress appraisals. It should be emphasized that Type D personality is a normal personality trait classification; this scale is not intended to be used as a deterministic screening tool for turnover risk, nor to inform discriminatory employment decisions.

Work system optimization aligned with primary prevention principles: Consistent with the primary prevention concept of the Occupational Stress Process Model, which identifies reducing exposure to stressors as a fundamental strategy to mitigate negative occupational outcomes, community health service centers and health administration departments may explore sustainable optimization of GPs’ workflow, streamlining of unnecessary paperwork and administrative tasks, rational allocation of workloads, improvement of compensation incentives and career development systems, and enhancement of GPs’ work autonomy and professional social recognition. These strategies are hypothesized to reduce exposure to objective occupational stressors at the source for all GPs, and may also reduce the environmental context that correlates with amplified stress perception among individuals with TDP traits, pending validation in future research.

Targeted, voluntary psychological support: Exploratory, personalized support programs (e.g., cognitive behavioral training, stress coping skills workshops, social skills counseling) may be offered on a voluntary basis to support GPs in managing negative emotions and perceived occupational stress. Previous research has suggested that positive emotional writing (Michael A. Smith) and expressive language use [73] may be beneficial for individuals with high Type D traits; where feasible, hospitals may provide guided positive writing sessions, encourage voluntary expressive writing, and set up optional emotional regulation spaces for interested GPs.

All of the above strategies are proposed as exploratory directions based on the observed associations in this study and existing theoretical frameworks, and their effectiveness in reducing turnover intention among GPs requires rigorous validation in future longitudinal and interventional research.

### 4.9. Strengths and Limitations

The present study has several strengths. First, this is the first study to examine the statistical associations among Type D personality, perceived occupational stress, job satisfaction, and turnover intention within an integrated model in Chinese general practitioners. Second, the study provides new ideas for evaluating the relationships between Type D personality, perceived occupational stress, job satisfaction, and turnover intention. Third, the results of our study informed potential strategies for the human resource management of GPs, especially with Type D personality.

Nevertheless, several limitations should be acknowledged. First, all participants were recruited solely from community healthcare centers in Longhua District, Shenzhen. This single-region sampling strategy limits the generalizability of our findings to the entire population of general practitioners across China. Second, all variables were measured using self-reported questionnaires at a single time point, which may introduce common method bias. Although procedural safeguards (anonymity, random item order) and Harman’s single-factor test were applied to reduce and assess CMB, more robust statistical control methods were not used. Therefore, common method bias may have either inflated or suppressed the observed associations, a limitation that should be considered in result interpretation. Third, this study employed a cross-sectional design, which prevents us from establishing temporal order or causal inferences among Type D personality, occupational stress, job satisfaction, and turnover intention, causal inferences cannot be drawn from this study. Fourth, although we examined the intervening statistical associations of stress and satisfaction, other relevant intervening variables such as social support and coping styles were not included, which may limit the comprehensiveness of the associative explanatory model. Fifth, some unmeasured confounders, such as physical health status, family factors, and work–family conflict, were not adjusted for in the analysis. Future studies should adopt longitudinal or experimental designs, adopt multisite sampling, include additional psychological mediators, and use multiple data sources to reduce common method bias, improve causal inference, and enhance generalizability.

## 5. Conclusions

In summary, the current findings indicate that general practitioners with Type D personality report a higher tendency of turnover intention, and this association is statistically accompanied by elevated perceived occupational stress and diminished job satisfaction. Within the framework of the hypothesized sequential model, these two variables account for a substantial proportion of the covariance between Type D personality and turnover intention. Hence, managers of Chinese community healthcare centers may prioritize interventions to reduce perceived occupational stress and improve job satisfaction, particularly for GPs with TDP. These strategies may be potentially useful to lower turnover intention and sustain a stable primary healthcare team, pending evidence from future longitudinal studies.

## Figures and Tables

**Table 1:** Comparisons of turnover intention among different socio-demographics.

Characteristics	N (%)	Turnover Intention (SE)	t/F	*p*-Value
Gender				
Male	161 (42.0)	1.57 (0.79)		
Female	222 (58.0)	1.67 (0.79)		
Age			1.313	0.270
≤30	85 (22.2)	1.69 (0.69)		
31–35	223 (58.2)	1.64 (0.87)		
≥36	75 (19.6)	1.50 (0.63)		
Marital status			0.246	0.620
Married	268 (70.0)	1.58 (0.78)		
Others	115 (30.0)	1.74 (0.81)		
Qualification			0.027	0.973
College or below	17 (4.4)	1.67 (0.77)		
Undergraduate	277 (72.3)	1.63 (0.81)		
Postgraduate	89 (23.2)	1.62 (0.73)		
Professional title				
Junior	75 (19.6)	1.80 (0.90)	4.77	<0.01
Middle	275 (71.8)	1.62 (0.78)		
Associate senior/Senior	33 (8.6)	1.30 (0.44)		
Work Hours			0.34	0.711
<8 h	70	1.63 (0.93)		
8–10 h	231	1.60 (0.75)		
Salary			3.95	<0.01
<15,000	73 (19.1)	1.74 (0.93)		
15,000–20,000	224 (58.5)	1.67 (0.81)		
20,001–30,000	73 (19.1)	1.47 (0.58)		
>30,000	13 (3.4)	1.08 (0.28)		

Note: Work Hours means average daily working hours; Salary means average monthly salary level.

**Table 2:** Association between Type D personality, perceived occupational stress, job satisfaction and turnover intention (B ^1^ and standard error).

	TDP	POS	JS	TI
TDP	1	0.30 (0.04) ***	−0.24 (0.03) ***	0.71 (0.08) ***
POS		1	−0.37 (0.04) ***	0.51 (0.06) ***
JS			1	−0.51 (0.05) ***
TI				1

^1^ Adjusted for gender, age, education, professional title, marital status, salary, and working hours. Note: POS: perceived occupational stress; JS: job satisfaction; TDP: Type D personality; TI: turnover intention; ***: *p*-value < 0.001.

**Table 3:** Decomposition of total covariance into direct and specific indirect associations in the hypothesized sequential model.

Path		(95% CI)	*p*-Value	Proportion of Model-Implied Covariance (%)
dir	TDP ⟶ TI	0.357 (0.196–0.536)	<0.01	69.32
ind	-	0.158 (0.053–0.282)	<0.01	30.68
Stdind b	TDP ⟶ POS ⟶ TI	0.089 (0.008–0.200)	<0.05	17.28
Stdind c	TDP ⟶ JS ⟶ TI	0.051 (0.002–0.109)	<0.05	9.90
Stdind d	TDP ⟶ POS ⟶ JS ⟶ TI	0.018 (0.001–0.048)	<0.05	3.50
total	-	0.515 (0.406–0.618)	<0.01	100.00

Note: POS: perceived occupational stress; JS: job satisfaction; TDP: Type D personality; TI: turnover intention.

## Data Availability

The data presented in this study are available on request from the corresponding author; they are not publicly available due to the ethical and privacy restrictions imposed by the relevant ethics committee.

## Abbreviations

The following abbreviations are used in this manuscript:

TDP	Type D personality
GP	General practitioner
POS	Perceived occupational stress
JS	Job satisfaction
TI	Turnover intention
DS-14	The Type D scale-14
PSS-14	Perceived Stress Scale-14
SEM	Structural equation modeling
